# Prediction of Heat Transfer and Fluid Flow Effects on Entropy Generation in a Monolithic Catalytic Converter Using Large-Eddy Simulation

**DOI:** 10.3390/e24050602

**Published:** 2022-04-26

**Authors:** Yongxiang Li, Luis Felipe Rico Cortes, Hardy Hamel, Kaushal Nishad, Luigi Biondo, Florian Ries

**Affiliations:** 1 Reactive Flows and Diagnostics, Technical University of Darmstadt, 64287 Darmstadt, Germany; felipe.rico@stud.tu-darmstadt.de (L.F.R.C.); hamel@rsm.tu-darmstadt.de (H.H.); nishad@ekt.tu-darmstadt.de (K.N.); biondo@rsm.tu-darmstadt.de (L.B.); ries@ekt.tu-darmstadt.de (F.R.); 2 Energy and Power Plant Technology, Technical University of Darmstadt, 64287 Darmstadt, Germany

**Keywords:** catalytic converter, large-eddy simulation, entropy production, Darcy–Forchheimer relation, flow laminarization

## Abstract

In the present work, heat transfer and fluid flow and their effects on entropy generation in a realistic catalytic converter of a Lada Niva 21214 vehicle are studied using large eddy simulation. At first, the pressure drop over the catalytic converter is measured for dry air at constant temperature (T=298 K), different volumetric flow rates, and extrapolated to large volumetric flow rates for dry air (T=298 K) and for the exhaust gas under realistic engine conditions (T=900 K) using the Darcy–Forchheimer relation. Then, coupled heat and fluid flow phenomena inside the catalytic converter are analyzed for nonreacting isothermal conditions and nonreacting conditions with conjugate heat transfer by using the large-eddy simulation. The predicted pressure drop agrees well with the measured and extrapolated data. Based on the obtained numerical results, the characteristic flow features are identified, namely: the impinging flow with stagnation, recirculation, flow separation and laminarization within the fine ducts of the monolith, which depends on the heat transfer through temperature-dependent thermophysical properties of exhaust gas. Moreover, due to high-velocity gradients at the wall of the narrow ducts in the monolith, entropy production by viscous dissipation is observed predominantly in the monolith region. In contrast, entropy production due to heat transport is relatively small in the monolith region, while it overwhelms viscous dissipation effects in the pipe regions.

## 1. Introduction

Modern automobiles powered by conventional internal combustion engines (ICEs) are subjugated to two major challenges: firstly, to comply with the increasingly stringent emission legislation, and secondly, to have the lowest possible carbon footprint by operating in a manner of best fuel efficiency. These challenges are being addressed either by replacing the existing power-train in battery electric vehicles (BEVs) or by operating ICEVs with so-called carbon-neutral fuels such as: bio-fuels or low carbon fuels such as hydrogen, natural gas, liquid petroleum gas, methanol, and synthetic fuels such as E-fuels, etc. Moreover, the complete replacement of traditional power-trains (ICEVs) by battery-operated engines (BEVs) is not foreseeable in the near future, rather, multiple power-trains will coexist [1], with more and more focus on the usage of carbon-neutral fuels on existing power-trains [2,3,4,5]. Thereby, it is still imperative to enhance incylinder technology, also for the new fuels, together with the deployment of more advanced exhaust gas after-treatment systems (EGAS). This way, a definite incentive is associated with the development of an advanced EGAS to meet the prescribed existing and future emission norms.

A crucial part of an EGAS is the catalytic converter, in which the harmful species in the exhaust gas are converted or reduced into less-toxic pollutants. In general, a modern catalytic converter consists of ceramic or metal monoliths, which has a honeycomb structure with channel densities of 64–124 cells/cm${}^{2}$, and the catalytic substance is distributed on the channel surface in porous form [6]. Thus, a large interface between exhaust gas and the catalytic substance is ensured by such a structure and an enhanced chemical reaction can be expected for effective reduction of pollutants. Since the last few decades, the catalytic converter has been widely investigated at the atomic level to understand the influence of various catalytic substances (i.e., Pt, Pd, Rh, etc.) on the oxidation and/or reduction efficiency of EGAS [7,8], understanding the complex and relevant chemical reaction kinetics [9,10], analyzing the process parameters on EGAS performance [11] or in designing the waste heat recovery system to utilize the thermal energy of the exhaust gas [12].

With regard to the numerical modeling and design optimization of EGAS, computational fluid dynamics (CFD)-based analysis is becoming a popular tool, since it can provide a comprehensive and macroscopic understanding of the coupled thermal and fluid flow dynamics inside the monolith. Additionally, it has the potential to reduce the overall designing cost and product life cycle. However, development of CFD-based numerical models of such a complex system itself is a challenging task, which requires careful consideration of numerical models at the individual component level. Focusing on the monolith of the catalytic converter, many studies have been carried out using the volume-averaging Navier–Stokes (VANS) methodology in the context of the Reynolds-averaged Navier–Stokes (RANS) model, in which the monolith is treated as a continuous porous medium, while the flow is resolved in a time-averaging manner [9,13,14]. The combination of VANS and RANS is able to provide a macroscopic view of the flow in the catalytic converter with low computational cost and simulation time. However, it does not allow a detailed understanding of the highly transient coupled heat and fluid flow phenomena taking place inside the monolith. In particular, the VANS/RANS approach experiences serious drawback while dealing with the transient evolution of velocity, pressure, and temperature fields with their respective fluctuations in the narrow monolith channels. Therefore, a better understanding of the coupled heat and fluid flow phenomena in the catalytic converter can be achieved only by resolving the heat and fluid flow structures also in the individual monolith channel, as pointed out recently in the work of Cornejo’s group [15]. They carried out simulations with different channel shapes to study their influence on the pressure drop across the monolith. Additionally, they analyzed the influence of upstream turbulence on the pressure drop in the catalytic converter based on the large-eddy simulations (LES) of a single monolith channel [16]. Thus, they demonstrated the importance of resolving the honeycomb structure of monolith and the applicability of the LES-based investigation of the complex EGAS.

As already pointed out in previous paragraphs, the catalytic converter features a complex and coupled thermofluid flow phenomena, making the design and process optimization a challenging task. In this aspect, by following the second law of thermodynamics, the entropy-generation-based irreversibility analysis of such a system can be a useful tool for both detailed understanding of the underlying physical phenomena and in identifying the key parameters for efficient operation of the EGAS. Such analysis is essentially based on the fact that a reduction in the thermodynamic efficiency of a thermal device is essentially associated with increase in the entropy production of this system, also known as entropy generation [17,18]. Thereby, the analysis based on entropy generation minimization (EGM) can be readily adopted, first to characterize the real industrial systems such as cryogenics, heat transfer, storage, solar energy conversion, power plants, refrigeration plants, etc., and then to optimize them for the most energy-efficient operations [19,20]. As demonstrated also in our previous contributions [21,22], an analysis based on entropy generation is a suitable approach to provide deeper insight about the coupled thermofluid flow processes, identify specific flow features, and choose the optimal operating point with minimum energy lost. A detailed description of entropy generation analysis, in particular in the context of LES, can be found in [21] and elsewhere. Several investigations based on entropy generation for different technical configurations and physical processes have been reported [18,22,23,24,25,26,27,28,29,30,31,32,33]. However, a study based on the entropy generation analysis for the realistic automobile catalytic converter has not yet been reported.

The lack of numerical investigation on the heat and turbulent flow in the catalytic converter using LES, especially to resolve the heat and fluid flow in all channels in the monolith, together with the absence of a study based on the entropy generation for such configuration, provides motivation to the present study. Thereby, the objective of this work is to propose the methodology for the prediction of heat transfer and fluid flow effects on entropy generation in a realistic automobile monolithic catalytic converter using LES. Notice that chemical reaction is not considered in this work and left for the future research. In the present work, the thermofluid dynamics in a realistic three-way catalytic converter of a Lada Niva 21214 with/without (conjugate) heat transfer is studied using LES methodology. To validate the applied numerical method, experimental measurements were also carried out, in which the pressure drop between the inflow and outflow of the monolith is measured for different volumetric flow rates. The present work is organized as follows. The applied numerical methods is outlined in Section 2. The geometry and operating conditions of the catalytic converter, as well as the numerical and experimental setup are described in Section 3. In Section 4, the results are presented and discussed in detail. Finally, the main findings of this study are highlighted in Section 5.

## 2. Large-Eddy Simulation

In the present numerical study, the solution domain consists of two parts, namely a fluid and a solid domain. In accordance with the procedure described in our previous works [33,34], the fluid part is governed by the balance equations for incompressible Newtonian fluid flow with variable physical properties and Fourier heat transport, while in the solid part only the energy equation is solved due to nonexistence of any flow. Thereby, in the context of LES, the low-Mach number (Ma<0.3) formulation for the balance laws of mass, momentum, and energy are employed and given as (see [35,36]):

(1) $$\frac{\partial \bar{\rho }}{\partial t}+\frac{\partial }{\partial x_{i}}\left(\bar{\rho }{\tilde{U}}_{i}\right)=0,$$

(2) $$\frac{\partial \bar{\rho }{\tilde{U}}_{i}}{\partial t}+\frac{\partial }{\partial x_{i}}\left(\bar{\rho }{\tilde{U}}_{i}{\tilde{U}}_{j}\right)=-\frac{\partial \bar{P}}{\partial x_{i}}+\frac{\partial }{\partial x_{j}}\left(\left(\bar{\mu }+\bar{\rho }\nu_{sgs}\right)\left(\frac{\partial {\tilde{U}}_{i}}{\partial x_{j}}+\frac{\partial {\tilde{U}}_{j}}{\partial x_{i}}-\frac{2}{3}\frac{\partial {\tilde{U}}_{k}}{\partial x_{k}}\delta_{ij}\right)\right),$$

(3) $$\frac{\partial \bar{\rho }\tilde{h}}{\partial t}+\frac{\partial }{\partial x_{i}}\left(\bar{\rho }{\tilde{U}}_{i}\tilde{h}\right)=\frac{\partial }{\partial x_{j}}\left(\left(\frac{\bar{\lambda }}{{\bar{c}}_{p}}+\frac{\nu_{sgs}}{Pr_{sgs}}\right)\frac{\partial \tilde{h}}{\partial x_{j}}\right),$$

where $\bar{\left(•\right)}$ and $\tilde{\left(•\right)}$ are the filtered and Favre-filtered quantities, respectively. ρ expresses the mass density, $U_{i}$ the velocity, *h* the sensible enthalpy, *P* the mechanical pressure, μ the molecular viscosity, λ the thermal conductivity, $c_{p}$ the isobaric heat capacity of the exhaust gas, $\nu_{sgs}$ is the subgrid-scale viscosity, and $Pr_{sgs}$ is the subgrid-scale Prandtl number. Regarding the modeling of subgrid-scale momentum transport, the Sigma model (σ-model) proposed by [37] is applied, while the linear thermal diffusivity model with constant subgrid-scale Prandtl number of $Pr_{sgs}=0.7$ is employed to model the unresolved heat flux.

For the solid part, the classical heat equation is valid, and reads:

(4) $$\begin{array}{c} \frac{\partial T^{s}}{\partial t}=\frac{\partial }{\partial x_{i}}\left(\alpha^{s}\frac{\partial T^{s}}{\partial x_{i}}\right). \end{array}$$

Here, $T^{s}$ represents the temperature and $\alpha^{s}$ the thermal diffusivity of the solid domain. The solid and fluid regions are coupled via a thermal interface, where the temperature and the heat flux of both phases have to be equal, which leads to the following constraints at the fluid–solid interface:

(5) $$\begin{array}{c} T^{s}=T^{f}\;\rho^{f}c_{p}^{f}\alpha^{f}\frac{dT^{f}}{dn}=\lambda^{s}\frac{dT^{s}}{dn}, \end{array}$$

where the terms with superscript ${\left(\cdot \right)}^{f}$ and ${\left(\cdot \right)}^{s}$ represent the quantities in the fluid and solid region, respectively. The thermal conductivity of the solid region is given by $\lambda^{s}=\rho^{s}c_{p}^{s}\alpha^{s}$, and *n* is the direction normal to the solid surface.

In this work, the working fluids, namely the air and the exhaust gas, are treated as homogeneous single component Navier–Fourier gases. Thereby, the thermodynamic and transport properties are calculated by means of seven-coefficient NASA polynomials [38], and transport properties are approximated based on Sutherland’s formula [39]. The material properties of the air and exhaust gas are evaluated using the material laws of the mixture. A detailed description about the adopted methodology can be found in [36]. The species composition and the thermodynamic properties of the exhaust gas are summarized in Appendix A.

## 3. Test Case: The Catalytic Converter

In this study, the coupled turbulent heat and fluid flow within a realistic three-way catalytic converter of a Lada Niva 21214 are investigated. The pressure drop of the catalyst is determined by experiment under nonreacting isothermal conditions and for different flow rates. These measurements are used to derive a Darcy–Forchheimer relation for the catalyst and to validate the numerical simulation. Then, the validated numerical approach is used to analyze nonreacting heat and fluid flow of exhaust gas within the catalytic converter under realistic operating conditions. The experimental and numerical setups applied in the present work are described in the following.

### 3.1. Experimental Setup

**Figure 1:**
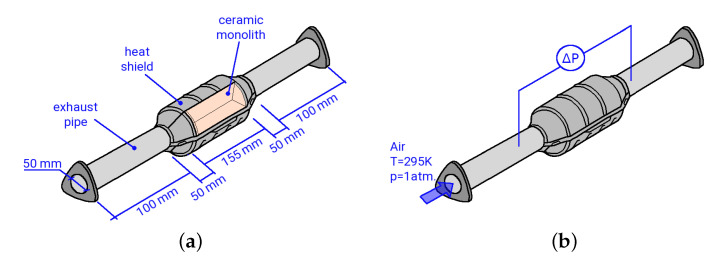
Illustration of the exhaust after-treatment catalytic converter; (**a**) assembly and dimensions; (**b**) measurement locations.

An illustration of the experimental test rig of the catalytic converter is depicted in Figure 1. A stream of dry air (T=295 K, p=1 atm) flows through the catalytic converter. The catalytic converter consists of a ceramic monolith at the center, which has a honeycomb structure with 12,468 small square channels, each with a dimension of 0.7 mm × 0.7 mm × 155 mm. Notice that an unwashed catalytic converter was used in the present study. The pressure drop between the inlet and outlet of the catalyst is measured by using a differential pressure sensor (differential pressure transmitters PD-23 of Keller AG) at constant volumetric flow rates of 10.4 m${}^{3}$/h to 139.6 m${}^{3}$/h. Therefore, the flow rates are controlled by means of a thermal mass flow controller (Bronkhorst EL-FLOW Select F-203AV) for precision tuning of the flow. The bulk of the flow is provided separately and measured by a thermal mass flow meter (Bronkhorst IN-FLOW F-116AI). Notice that a perforated plate is located ten pipe diameters upstream of the monolith. This perforated plate serves as a turbulence-generating grid and ensures that the turbulent flow is fully developed at the entrance of the catalytic converter (see [36]).

### 3.2. Numerical Setup

**Figure 2:**
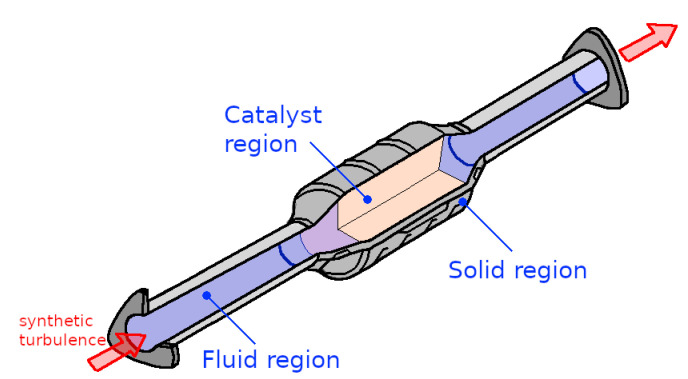
Numerical treatment of the catalytic converter configuration: domain coupling fluid/solid/catalyst regions.

In the numerical study, the honeycomb structure of the monolith with 12,468 small ducts is fully resolved by means of the numerical grid. Figure 2 shows the coupled fluid and solid regions that are solved in this numerical study. The catalytic converter is essentially divided into: (i) two pipe flow regions, (ii) a catalyst region which includes the honeycombs structure, and (iii) the solid region. These regions are coupled via interfaces with each other. The solid region is modeled using solid properties of iron ($\rho^{s}$ = 7853 kg/m${}^{3}$ and $\kappa^{s}$= 80.2 W/mK), and the monolith is modeled by solid properties of a representative ceramic ($\rho^{s}$ = 1500 kg/m${}^{3}$ and $\kappa^{s}$= 2 W/mK); the insulation packing is represented by a representative insulation material ($\rho^{s}$ = 1840 kg/m${}^{3}$ and $\kappa^{s}$= 0.3 W/mK).

Regarding the boundary conditions, the no-slip velocity boundary condition is applied to the interface between fluid body and solid body, while coupled thermal boundary conditions are used for the temperature (see Equation (4)). Additionally, both the near-wall region and the flow in the catalyst region are fully resolved by the numerical grids. At the inlet, a realistic turbulent inflow condition is generated using the digital filter approach as proposed by [40]. The unresolved momentum transport is modeled by using the σ-model [37], while the linear diffusivity model with $Pr_{sgs}=0.7$ is used to model the unresolved heat transport. The thermodynamic properties of the applied exhaust gas is described in Appendix A. Thereby, in case of hot exhaust gas flow with constant inflow temperature of T=900 K, a uniform heat transfer coefficient ($h_{0}=152\frac{W}{m^{2}K}$) and ambient temperature T=298 K are specified for the outer wall of the solid region, representing the heat transfer to the ambient. In case of cold air flow, isothermal conditions of T=298 K without any wall heat transfer is assumed.

**Figure 3:**
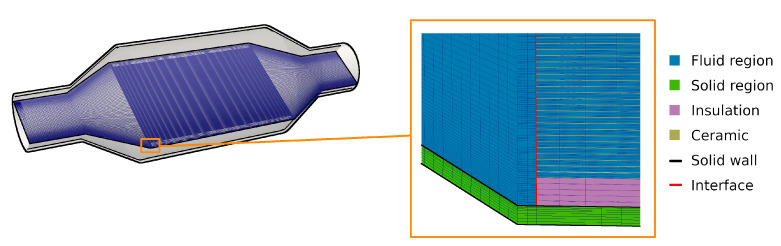
The representation of applied numerical grids for solid and fluid regions that are coupled via interfaces.

In order to analyze the grid dependency of the numerical results, three numerical grids with different spatial resolutions are employed (see Table 1), denoted here as coarse, medium, and fine. The schematic representation of mesh for different regions with respective interfaces are provided in Figure 3. In the first refinement step (from coarse to medium grid size), the solid and the fluid domains are refined, while in the second refinement step only the fluid region is refined. Therefore, each channel in the monolith is discretized in the fluid domain by (h×w×l)=(3×3×16) (coarse), (6×6×20) (medium), and (12×12×24) (fine) cells, respectively. This results in a maximum wall distance of $y^{+}<3.2$ at the duct walls of the monolith for the finest spatial resolution, while $y^{+}$ is smaller than one in the rest of the computational domain. Furthermore, second order numerical schemes are applied in space and time for the numerical simulations, and the maximum CFL number was set to $CFL_{max}<0.8$. The simulation results are assumed to be converged in case the relative residual is smaller than $\varepsilon <10^{-3}$. This was checked for each time step. The simulation cases investigated in this study are listed in Table 2. Notice that for the highest spatial resolutions (case 3, 6, 9, and 12) approximately 0.5 million CPUh are required to solve 0.1 s of physical time for each case.

## 4. Results and Discussion

In this section, the obtained numerical results for nonreacting turbulent heat and fluid flow characteristics of an exhaust-after-treatment system of a Lada Niva 21214 are discussed in detail. First, in the case of isothermal flow conditions (cases 1–9 of Table 2), predictions of the pressure drop are compared with the experimental data. Then, heat and fluid flow features inside the monolith are analyzed under nonreacting nonisothermal conditions, and finally, entropy generation rates due to viscous dissipation and heat transport within the catalyst are characterized (cases 10–12 of Table 2). Notice that the heat and fluid flow inside the catalyst and the entropy generation rates are only analyzed using numerical methods since such quantities are obviously very difficult to quantify experimentally inside a three-way catalytic converter.

### 4.1. Comparison with Experimental Data (Case 1–12)

**Figure 4:**
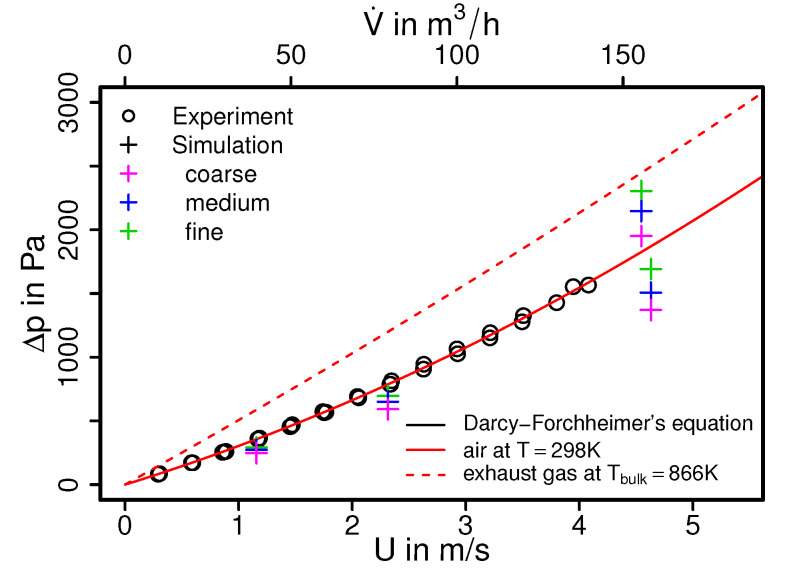
Pressure drop characteristic of a three-way catalytic converter of a Lada Niva 21214. *U* denotes the bulk velocity inside the monolith.

In an automobile engine, the exhaust back pressure is a key design parameter and plays a determining role in efficient engine operation, while the affordable back pressure of an engine largely depends of the pressure drop across various components along the exhaust after-treatment circuits. In this regard, Figure 4 shows the comparison of measured and simulated mean pressure drops as a function of the volumetric flow rates for a three-way catalytic converter employed in Lada Niva 21214. The experimental data is presented in a black circle and simulation data are shown as crosses, while the red solid line denotes a best fit of the measured pressure drop based on the Darcy–Forchheimer law [41]. The dashed red line shows the corresponding Darcy–Forchheimer law for the hot exhaust gas (T=900 K). Additionally, the pressure drop between the inflow and outflow side of the catalytic converter can be modeled very accurately by means of the Darcy–Forchheimer equation. For the present catalyst the Darcy–Forchheimer equation reads [41]:

(6) $$-\frac{\Delta P}{\Delta l}=\frac{\mu }{K}U+\frac{\rho }{k_{2}}U^{2},$$

with the fitted coefficients *K* = 1.7077 × 10${}^{-8}$ and $k_{2}=0.01077$. The first term on the RHS describes the pressure drop due to friction, while the second term is related to turbulent dissipation. By means of this equation, the experimental data can be extrapolated to the case with higher volumetric flow rates and nonisothermal conditions. Notice that the quantities for hot exhaust gas are evaluated based on the averaged bulk temperature in the monolith obtained by the numerical simulation with fine grid resolution.

As it can be seen in Figure 4, predicted mean pressure drops obtained from the simulation compared well with the experimental data and also with the derived Darcy–Forchheimer relation. This holds true for both cold air flow and hot exhaust gas flow. Therefore, good agreements are achieved for all flow rates under consideration. However, it can be observed that the predicted pressure drops from the simulation are slightly lower than the measured ones. This is mostly visible in the results from the cases with coarse grid and higher flow rates.

Besides the total pressure drop of the catalytic converter, it is also of interest to compare predicted skin friction at the duct walls in the monolith, which is directly connected to entropy production related to viscous dissipation. In the case of fully developed laminar duct flow, the friction coefficient can be calculated analytically as:

(7) $$c_{f}=\frac{2\tau_{w}}{\rho u_{b}^{2}}=\frac{2Po}{Re},$$

with a Poiseuille number of Po=7.1135 for a duct with square cross-section shape (see [42]). This leads to a classical value of $c_{f}^{th.}=0.0492$ for case 9 with a Reynolds number of Re=289 of the duct flow at the center of the monolith. The friction coefficient predicted by the LES simulation is found to be $c_{f}^{sim.}=0.0421$, which is slightly lower than the analytical value.

In summary, deviations of LES predictions with the finest grid resolution are relatively small (∼10%) considering such a complex test case of a realistic catalytic converter. This allows us to conclude that the present numerical approach is suitable to predict the key operation parameters of the catalytic converter. It can therefore be used for further analyses of the heat and fluid flow properties and entropy production inside the catalytic converter.

### 4.2. Predictions of Heat and Fluid Flow Features (Case 9 and 12)

**Figure 5:**
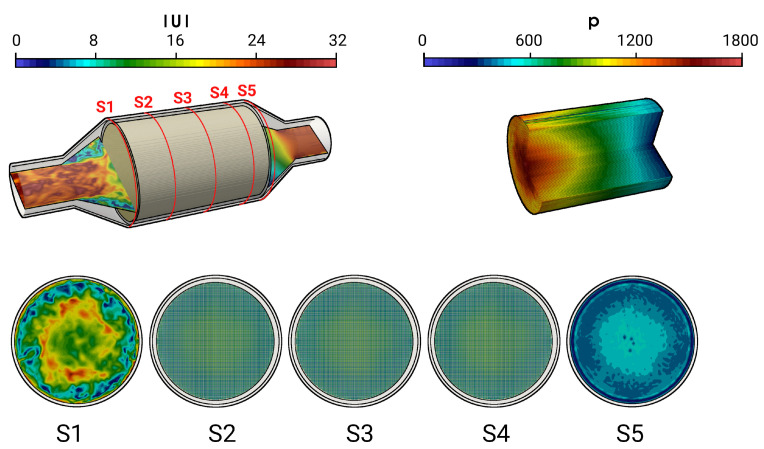
Instantaneous velocity field in the catalytic converter and instantaneous pressure field in the monolith along with velocity profile in various cross sectional planes S1–S5.

Along with other factors, the efficiency of a catalytic converter is largely determined by how uniformly the flow and thermal fields are distributed upstream to the monolith. This allows the effective utilization of available surface area in the monolith for the intended purpose of particulate filtration, oxidation, reduction, etc. In this context, Figure 5 shows a snapshot of the instantaneous magnitude velocity field of the isothermal case with $\dot{V}$ = 160.0 m${}^{3}$/h (case 9) together with the velocity distribution at several cross-sections (S1–S5) and the pressure distribution along the the monolith channel (see Figure 5, top right).

**Figure 6:**
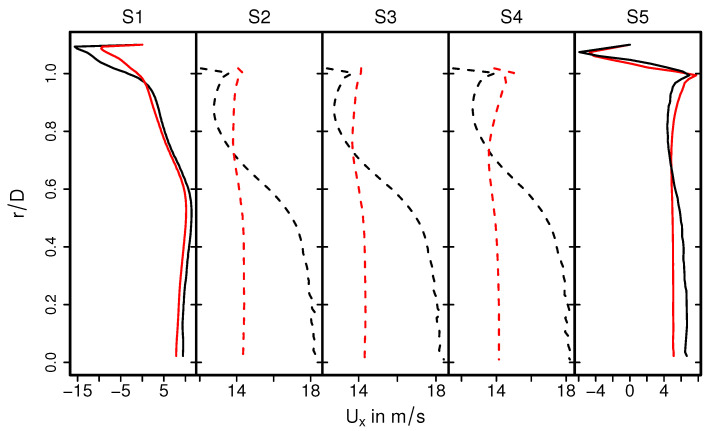
Velocity distributions in the main flow direction at different cross-sections for isohermal case (black lines) and nonisothermal nonreacting case (red lines). The dashed lines represent the velocity profile inside the monolith region.

**Figure 7:**
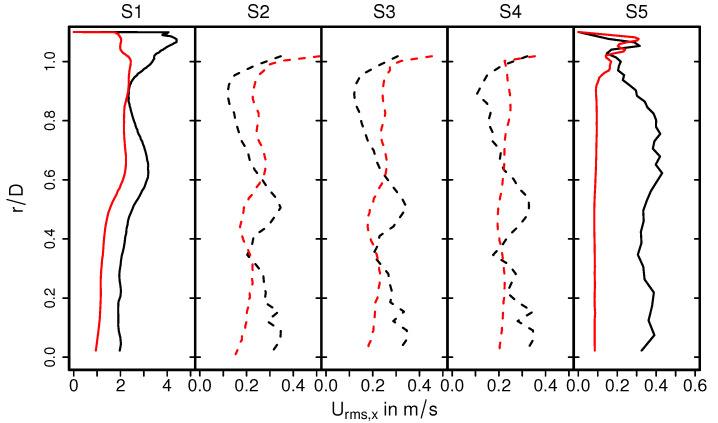
Comparison of rms velocity fields on selected planes.

A highly turbulent intake jet can be observed that impinges on the monolith, resulting in recirculation and separation flow in the front of the monolith. Therefore, the pressure around the stagnation point is high and decreases rapidly in the flow direction. Traveling further downstream, the flow gets laminarized inside the monolith and the pressure distribution becomes homogeneous in the radial direction. It is also interesting to observe that even the velocity is small at the stagnation region; the velocity inside the monolith is high around the center line and decreases in radial direction. This resultant velocity profile inside the monolith remains constant further downstream (see the velocity profile in sections S2–S4). This observation is analyzed quantitatively in Figure 6 and Figure 7 for the predicted mean and root mean square (rms) velocities in longitudinal direction, respectively, at the cross-sections S1–S5. Here, *D* is the diameter of the inlet pipe and *r* is the radial coordinate that points to the center (r/D=0 is located at the duct or monolith center line). The quantities of the isothermal case (case 9) are plotted with black lines, while for the nonreacting nonisothermal case (case 12) they are plotted with red lines. Notice that dashed lines in the cross-sections S2–S4 represent the velocity profile inside the monolith region.

It can be seen in Figure 6 that the mean velocity profiles out of the monolith (S1, S5) are not significantly influenced by the heat transfer. In contrast, mean velocity profiles for case 9 and case 12 differ significantly within the monolith. Therefore, the velocity profile in the nonreacting nonisothermal case is of uniform shape along the radial direction, while the velocity magnitude is maximal at center line (r/D=0) and decreases gradually in radial direction in case of isothermal conditions. These observations can be mainly attributed to the change in thermophysical properties, in particular the change in the viscosity of the exhaust gas due to temperature change. Moreover, these properties remained constant under isothermal conditions. In the case of velocity fluctuations, it is observed in Figure 7 that the rms velocity is high at the impinging region (S1) decreases rapidly in the monolith and remains small in the entire monolith. This holds true for both isothermal and the nonisothermal conditions, which allows us to conclude that the laminarization of the flow in the monolith is driven largely by fluid flow effects rather than heat transport phenomena. It should also be noted here that the rms velocity obtained for isothermal conditions is higher than that of nonisothermal conditions, especially along the impingement section highlighting the impact of increased viscosity due to higher temperature in the nonisothermal case.

**Figure 8:**
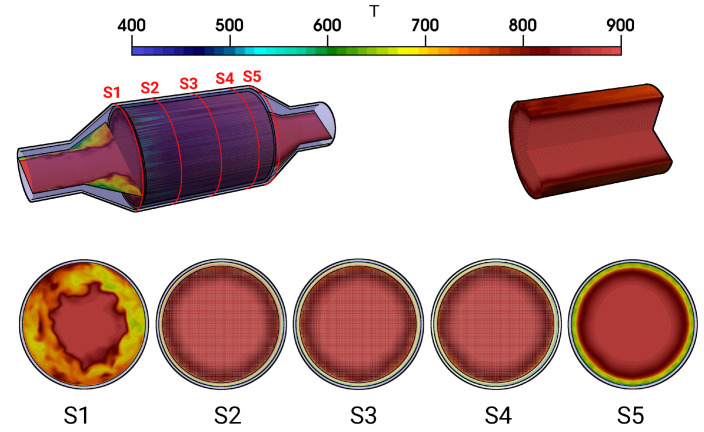
Instantaneous temperature fields in the catalytic converter and instantaneous pressure field in the monolith along with velocity profile in various cross-sectional planes S1–S5.

**Figure 9:**
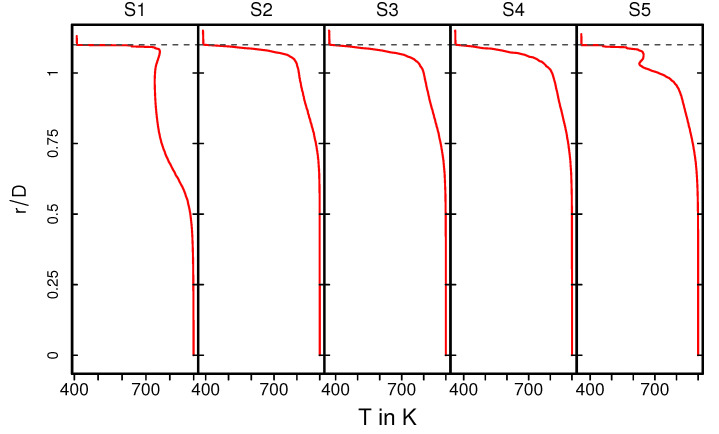
Profiles of mean temperature along the radial direction at various cross sections.

**Figure 10:**
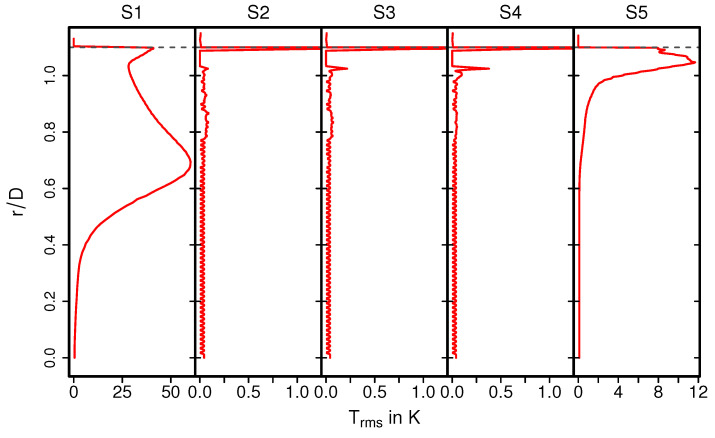
Profiles of rms temperature along the radial direction at various cross- sections.

The temperature distribution inside the catalytic converter is shown in Figure 8 (case 12) for the fluid regions, the solid parts, and for several cross-sections (S1–S5) (see Figure 8, bottom). Similar to the velocity field, the hot turbulent intake jet impinges on the front side of the catalyst. Therefore, the evolution of the thermal field inside the monolith appears to be very similar to that of the velocity field (see Figure 5). However, temperature gradients are significantly larger at the solid–fluid interface than at the fluid–ceramic interface. This holds true for all cross-sections in the monolith (see Figure 8, bottom (S2–S4)). The corresponding mean and rms temperature profiles along the radial direction are plotted in Figure 9 and Figure 10 for the cross-sections (S1–S5), respectively.

The exhaust gas temperature is relatively low upstream the monolith due to intense heat transfer caused by recirculation (see also Figure 9, S1). In contrast, inside the monolith, at the cross sections S2–S4, the exhaust gas is heated up due to heat transfer from the ceramic mass and the temperature profile is more homogeneous, especially around the core of the monolith. This tendency is maintained downstream to the monolith at section S5. Such temperature distribution is essentially responsible for the increased exhaust gas viscosity around the core region, while the viscosity is lower towards the solid–fluid interface region. This allows us to explain the homogeneous velocity profile for the nonreacting nonisothermal case, as observed in Figure 6. The rms temperature profile is shown in Figure 10. At S1, a significantly higher temperature fluctuations can be observed, while it is more or less negligible inside the monolith (S2–S4) and to certain extent also at S5. The higher temperature fluctuation is persistent along the solid–fluid interface at all cross-sectional locations (S1–S5) with negligible temperature variations in the solid region.

### 4.3. Predictions of Entropy Production Rates

Based on the observations in the previous section, it appears that heat and fluid flow phenomena inside the catalytic converter are extremely complex and are essentially a coupled heat and fluid flow process together with turbulence transition inside the monolith. To obtain further insight about such complex and interacting processes, entropy generation-based analysis is carried out next.

In turbulent fluid flow with heat transfer, the entropy production rates can be separated into two parts, namely entropy production rate by viscous dissipation $\Pi_{v}$ and, due to heat transfer, $\Pi_{q}$. According to [21,36], in the context of LES, the temporal averaged filtered entropy production rates $\langle {\bar{\Pi }}_{v}\rangle$, and $\langle {\bar{\Pi }}_{q}\rangle$ can be calculated as the sum of resolved and residual part as:

(8) $$\langle {\bar{\Pi }}_{v}\rangle =\underset{\langle {\bar{\Pi }}_{v}^{res}\rangle }{\underset{︸}{\langle \frac{\bar{\mu }}{\bar{T}}\left(\frac{\partial {\bar{U}}_{i}}{\partial x_{j}}+\frac{\partial {\bar{U}}_{j}}{\partial x_{i}}\right)\frac{\partial \bar{U_{i}}}{\partial x_{j}}\rangle }}+\underset{\langle {\bar{\Pi }}_{v}^{sgs}\rangle }{\underset{︸}{\frac{\langle \bar{\rho }\rangle }{\langle \bar{T}\rangle }\frac{{\langle \nu_{t}\rangle }^{3}}{\Delta^{4}C_{S}^{4}}}}$$

(9) $$\langle {\bar{\Pi }}_{q}\rangle =\underset{\langle {\bar{\Pi }}_{q}^{res}\rangle }{\underset{︸}{\langle \frac{\bar{\lambda }}{{\bar{T}}^{2}}\frac{\partial \bar{T}}{\partial x_{j}}\frac{\partial \bar{T}}{\partial x_{j}}\rangle }}+\underset{\langle {\bar{\Pi }}_{q}^{sgs}\rangle }{\underset{︸}{\frac{4\langle \bar{\rho }\rangle \langle {\bar{c}}_{p}\rangle \langle \nu_{t}\rangle }{3C_{OC}\pi^{4/3}C_{s}^{4/3}\langle Pr\rangle {\langle \bar{T}\rangle }^{2}}\langle \frac{\partial \bar{T}}{\partial x_{i}}\frac{\partial \bar{T}}{\partial x_{i}}\rangle }},$$

with $C_{OC}=1.34$ the Obukhov–Corrsin constant [43], $C_{S}$ the Smagorinsky coefficient [44], and Δ the filtered width.

**Figure 11:**
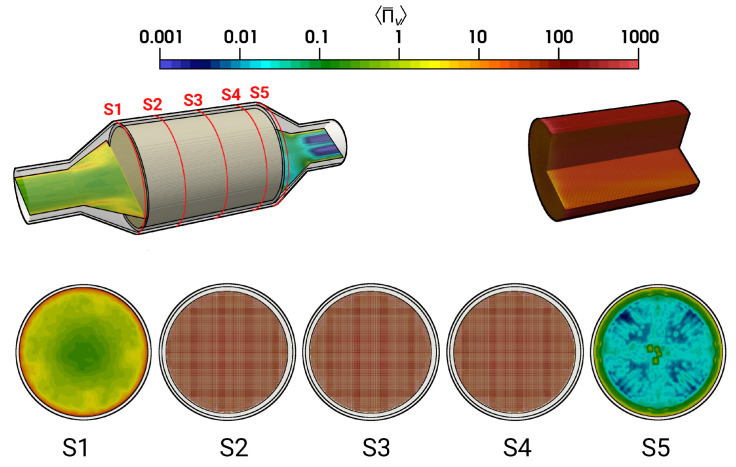
Time-averaged entropy production rate by viscous dissipation (case 9).

The calculated time-averaged entropy production rate related to viscous dissipation $\langle {\bar{\Pi }}_{v}\rangle$ within the catalytic converter is provided in Figure 11 for the isothermal test case (case 9). Therefore, a logarithmic color map is employed to visualize the wide range of entropy production scales.

**Figure 12:**
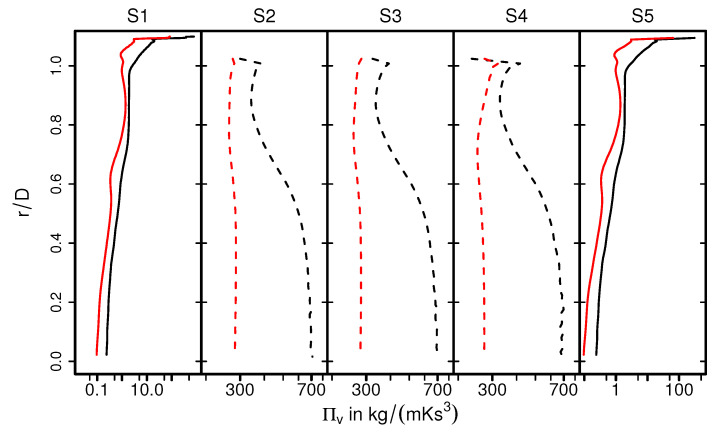
Entropy production rates by viscous dissipation $\Pi_{v}$ on selected planes for the isohermal case (black lines), and nonisothermal case (red lines). The dashed lines represent the quantities at the ceramic–fluid wall in the monolith.

As it is visible in Figure 11 (for case 9), for the section S1, the higher entropy production rates $\langle {\bar{\Pi }}_{v}\rangle$ are located predominantly close to the wall where a steep velocity gradient exists, and it decreases rapidly towards the core flow region. However, considerably higher entropy generation rate $\langle {\bar{\Pi }}_{v}\rangle$ is observed inside the monolith (S2–S4). This observation can be attributed to the strong turbulent dissipation due to wall-bounded flow along the narrow individual monolith channels, which ultimately results in flow laminarization. Moving further downstream to the monolith (at section S5), the $\langle {\bar{\Pi }}_{v}\rangle$ profile features a classical pipe flow characteristic. In order to analyze the impact of heat transfer, Figure 12 presents a comparison of $\langle {\bar{\Pi }}_{v}\rangle$ for isothermal (case 9) and nonisothermal (case 12) conditions. Notice that the dashed lines of S2, S3, and S4 represent values of $\langle {\bar{\Pi }}_{v}\rangle$ close to the ceramic wall. Outside the monolith (S1, S5), the distributions of $\langle {\bar{\Pi }}_{v}\rangle$ are very similar for isothermal and nonisothermal conditions. In contrast, values of $\langle {\bar{\Pi }}_{v}\rangle$ differ significantly for isothermal and nonisothermal conditions within the monolith (S2–S4). Therefore, values of $\langle {\bar{\Pi }}_{v}\rangle$ are significantly higher under isothermal conditons, which suggests that the irreversible laminarzation process is more intense for such conditions. Notice that the calculated $\langle {\bar{\Pi }}_{v}\rangle$ is higher in the core region of monolith under isothermal conditions (case 9, black dash line), implying the combined influence of carrier gas properties and flow profile (see Figure 6) on the entropy generation rate due to viscous dissipation.

**Figure 13:**
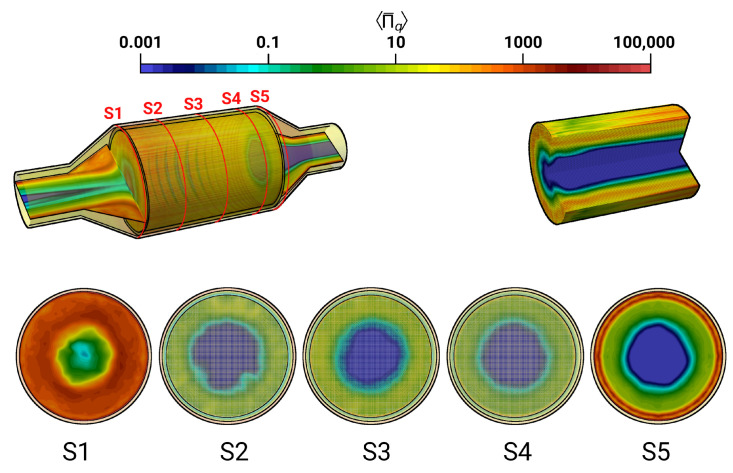
Time-averaged entropy production rate by heat transfer (case 12).

**Figure 14:**
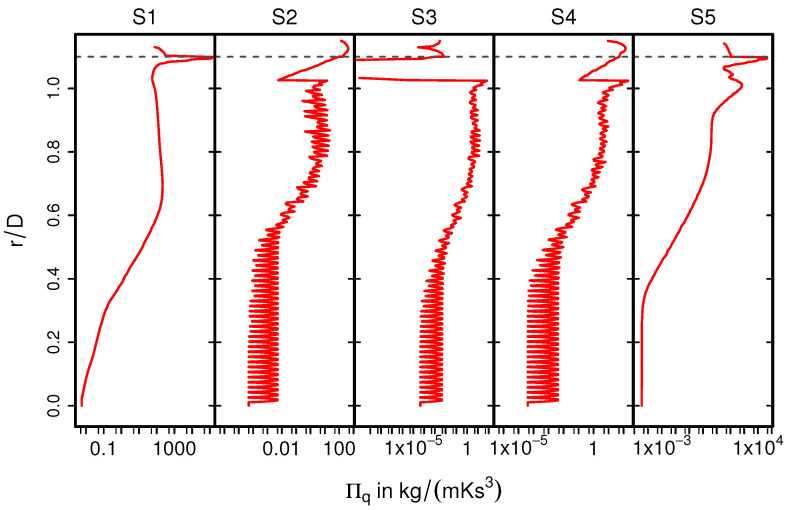
Entropy production rate by heat transfer $\Pi_{q}$ on selected planes (case 12).

Next, Figure 13 shows the time-averaged entropy production rates related to heat transport $\langle {\bar{\Pi }}_{q}\rangle$ for nonreacting nonisothermal conditions (case 12). The corresponding profiles of $\Pi_{q}$ are depicted in Figure 14. Due to the intense heat transfer at the impingement region (see Figure 13, S1 and Figure 14, S1), strong temperature gradients occurs in the vicinity of the solid walls, which results in high values of $\langle {\bar{\Pi }}_{q}\rangle$. In contrast, the value of $\langle {\bar{\Pi }}_{q}\rangle$ is considerably small inside the monolith (S2-S4), especially at the core of the monolith and less evenly distributed as compared with $\langle {\bar{\Pi }}_{v}\rangle$. Furthermore, at location S5 (see Figure 13 and Figure 14), the entropy generation rate is primarily due to strong temperature gradients.

To provide a more global perspective of entropy generation in the catalytic converter, the integral values of $\langle {\bar{\Pi }}_{v}\rangle$ and $\langle {\bar{\Pi }}_{q}\rangle$ for the monolith region and pipe regions are listed in Table 3 for both isothermal and nonisothermal cases under nonreacting conditions.

From Table 3, it can be concluded that entropy production by viscous dissipation ($\Pi_{v}$) occurs predominantly in the monolith region due to high-velocity gradients at the walls of the narrow monolith ducts, where a large amount of kinetic energy is dissipated through friction. This suggests that the laminarization inside the monolith is purely a fluid flow process. In contrast, irreversible thermal processes are more intense in the pipe regions, as the total value of $\Pi_{q}$ is quite high there. Furthermore, entropy production related to heat transport is approximately ten times higher than entropy production due to viscous dissipation for this specific catalytic converter.

## 5. Conclusions

In the present work, the pressure drop over a realistic catalytic converter of a Lada Niva 21214 vehicle was measured for dry air at T=298 K and different volumetric flow rates. Correlations were derived for isothermal conditions (dry air at T=298 K) and for realistic engine conditions (for exhaust gas at T=900 K) using the Darcy–Forchheimer law. The measured pressure drops were compared with large-eddy simulation results for different flow rates and working fluids showing good agreement. The validated numerical approach is then used to analyze the coupled thermal and fluid flow processes inside the catalytic converter under different operating conditions. Finally, entropy generation analysis is employed to identify the process irreversibility inside the catalytic converter under realistic engine conditions. Therefore, the following conclusions can be drawn from this work:

- The Darcy-Forchheimer equation is well suited to describe the pressure drop in this specific catalytic converter. Correlation equations based on the experimental data are provided.
- Important characteristic flow features are identified in the catalytic converter, namely the impinging flow with stagnation, recirculation, flow separation, and laminarization within the fine ducts of the monolith.
- The rms velocity decreases rapidly in the monolith, attributing to the flow laminarization process in the narrow monolith channels. This physical process is influenced by the heat transfer dynamics through temperature-dependent thermophysical properties as simulations with and without heat transfer testify.
- The entropy production by viscous dissipation ($\Pi_{v}$) occurs predominantly in the monolith region due to high-velocity gradients at the walls of the narrow monolith ducts. This suggests that the laminarization inside the monolith is purely a fluid flow process through temperature-dependent thermophysical properties.
- The entropy production rate due to heat transport $\Pi_{q}$ is relatively small in the monolith region, while it overwhelms viscous dissipation effects in the pipe regions.

## Figures and Tables

**Table 1:** Number of grid points used in the present numerical study.

	Coarse	Medium	Fine
solid part	1,499,560	2,348,408	2,348,408
fluid part	5,556,400	13,959,292	46,349,536

**Table 2:** The list of the test cases considered in the numerical study. (See Table 1 for numerical grids.) The Reynolds number $Re_{pipe}$ is specified for the pipe inflow and pipe diameter. $Re_{monolith}$ represents the Reynolds number inside the monolith channels.

Case	Grid	Flow Rate	Fluid	Temperature	Repipe	Remonolith
1	coarse	40.3 m${}^{3}$/h	air	298 K	15,921	73
2	medium	40.3 m${}^{3}$/h	air	298 K	15,921	73
3	fine	40.3 m${}^{3}$/h	air	298 K	15,921	73
4	coarse	79.8 m${}^{3}$/h	air	298 K	31,527	144
5	medium	79.8 m${}^{3}$/h	air	298 K	31,527	144
6	fine	79.8 m${}^{3}$/h	air	298 K	31,527	144
7	coarse	160 m${}^{3}$/h	air	298 K	63,213	289
8	medium	160 m${}^{3}$/h	air	298 K	63,213	289
9	fine	160 m${}^{3}$/h	air	298 K	63,213	289
10	coarse	160 m${}^{3}$/h	exhaust gas	900 K	10,020	46
11	medium	160 m${}^{3}$/h	exhaust gas	900 K	10,020	46
12	fine	160 m${}^{3}$/h	exhaust gas	900 K	10,020	46

**Table 3:** Entropy production rates related to viscous dissipation $\Pi_{v}$ and heat transport $\Pi_{q}$ integrated over the monolith and pipe flow regions.

	Monolith Region	Pipe Regions	Conditions
$\langle {\bar{\Pi }}_{v}\rangle$	182.877	5.104	isothermal
$\langle {\bar{\Pi }}_{v}\rangle$	102.886	3.112	non-isothermal
$\langle {\bar{\Pi }}_{q}\rangle$	82.221	1249.484	non-isothermal

**Figure A1:**
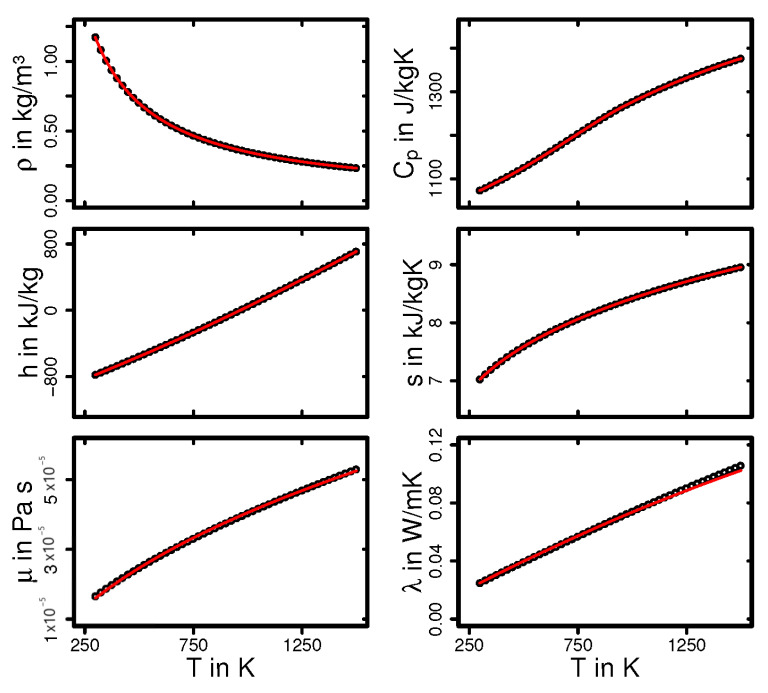
Comparison of the thermodynamic and transport properties calculated by the existing material laws and their coefficients (in circles) and calculated by the new material laws of exhaust gas, in which the coefficients are generated by least-square fits (in lines).

## Data Availability

Not applicable.

## Abbreviations

The following abbreviations are used in this manuscript:

ICEV	Internal Combustion Engine Vehicle
BEV	Battery Electric Vehicle
EGM	Entropy Generation Minimization
EGAS	Exhaust Gas After-treatment System
CFD	Computation Fluid Dynamics
VANS	Volume-Averaging Navier–Stokes
RANS	Reynolds-Averaging Navier–Stokes
LES	Large-Eddy Simulation

## Appendix A. Thermodynamic Properties of Applied Exhaust Gas

The species composition of the exhaust gas has a constant value according to [6] and summarized in Table A1. Instead of treating the exhaust gas as a mixture of different gases, it is considered as an homogeneous single component Navier–Fourier gas, whereby thermodynamic properties are calculated by means of seven-coefficient NASA polynomials [38] and transport properties are approximated based on Sutherland’s formula [39]. Therefore, the material laws for the properties of the exhaust gas are evaluated using the material laws of the mixture. The molar mass *M* of the presented exhaust gas is equal to 0.02863 kg/mol, and the density is calculated for a reference pressure at 1 atm. Using NASA coefficients, which are obtained by a least-square fit and listed in Table A2, the heat capacity $C_{p}$ (associated with $a_{1}-a_{5}$), sensible enthalpy *h* ($a_{6}$ ), and entropy *s* ($a_{7}$) are expressed as polynomial. The dynamic viscosity μ and the thermal conductivity λ are evaluated by the model of Sutherland with two coefficients $A_{Suth}$ = 1.5335 × 10^−6^ and $T_{Suth}=1$95.6627, respectively. A comparison of the fitted values and the theoretical values are depicted in Figure A1. An excellent agreement is observed; the presented coefficients are therefore applied in the simulation.

**Table A1:** Species composition of exhaust gas.

species [-]	$CO_{2}$	$H_{2}O$	$O_{2}$	$N_{2}$	CO
fraction [%]	12.3	13.8	0.7	72.3	0.9

**Table A2:** NASA coefficients of exhaust gas, generated by a least-square fit.

NASA Coefficient	$a_{1}$	$a_{2}$	$a_{3}$	$a_{4}$	$a_{5}$	$a_{6}$	$a_{7}$
T≤1000 K	3.48	7.08 × 10^−4^	−2.61 × 10^−7^	1.24 × 10^−9^	−7.77 × 10^−13^	−1.10 × 10^4^	4.15
T>1000 K	3.13	1.77 × 10^−3^	−5.91 × 10^−7^	9.04 × 10^−11^	−5.10 × 10^−15^	−1.10 × 10^4^	5.88
